# Impact of transesophageal echocardiogram findings on management of acute ischemic stroke: a retrospective single-institution cohort study

**DOI:** 10.1097/MS9.0000000000002838

**Published:** 2025-01-07

**Authors:** Sherif Eltawansy, Abbas Alshami, Paweł Łajczak, Sowmya Dandu, Renato Apolito

**Affiliations:** a Internal Medicine Jersey Shore University Medical Center, New Jersey, USA; b Cardiology Department, Jersey Shore University Medical Center, New Jersey, USA; c Medical University of Silesia, Poniatowskiego 15, 40-055 Katowice, Poland

**Keywords:** diagnostic, echocardiography, echocardiography, embolic stroke, imaging, ischemic stroke, patent foramen ovale, transesophageal, transthoracic

## Abstract

**Background:**

Physicians use transesophageal echocardiography (TEE) as part of the workup for acute ischemic stroke; however, its importance is controversial. While its clinical utility in this patient population may seem logical, based on current data and guideline recommendations, there is no empirical recommendation for using TEE over a transthoracic echocardiogram (TTE). This study aimed to provide an update on the TEE’s impact on the clinical outcomes of patients with acute stroke.

**Methods:**

We conducted a retrospective single-institution cohort study at a tertiary care hospital from June 2021 to August 2022 for patients admitted with new-onset ischemic stroke with or without hemorrhagic conversion who subsequently had a TEE completed at the same admission or after discharge. The primary outcome was the detection of management changes based on TEE results, while secondary outcomes included positive findings leading to embolic stroke, the feasibility of performing TEE as an inpatient versus outpatient procedure, and the TTE yield compared to TEE.

**Results:**

A total of 176 patients underwent TEE to investigate the cause of stroke. The mean age of those with positive TEE findings was 65.5 years versus 66 years for those with negative or irrelevant findings. Positive TEE patients were 47.7% female versus 45% in the negative finding group. Both age and sex comparisons did not yield statistically significant differences (*P*-values of 0.81 and 0.73, respectively). Patients with positive TEE findings accounted for 66 (37.5%), with the most common cardiac pathology being patent foramen ovale (PFO), followed by aortic atherosclerotic plaque. The transthoracic echocardiography (TTE) yield was much lower than that of TEE.

**Conclusion:**

In patients with newly diagnosed stroke, TEE provided high diagnostic values and impacted the management of 23.9 % of all patients who underwent the test. These findings support its continued use in diagnostic workups, with a higher diagnostic yield than the transthoracic approach.

## Introduction

Clinical investigation following an acute ischemic stroke involves an extensive diagnostic workup for the underlying reasons, as it is conducted to aid in selecting targeted secondary prevention therapies. Cardioembolic phenomena represent a significant source of ischemic stroke, accounting for approximately 20–30% of all cases^[1,2]^. The echocardiogram, with its two approaches, a transthoracic echocardiogram (TTE) and a transesophageal echocardiogram (TEE), is pivotal in exploring the causes of ESUS (embolic stroke of unknown source), which is a significant subset of cardioembolic strokes. These causes include patent foramen ovale (PFO), endocarditis, intracardiac masses (tumor or thrombus), and aotric atheromatous plaques^[3]^. The 2021 guidelines from the American Heart Association (AHA) currently endorse using TEE in the cardioembolic patient population, with a class IIb (C-LD) recommendation^[4]^. Different studies, including systematic literature review articles, strongly emphasize the echocardiography’s role, with both modalities, in acute stroke patients. In addition, evidence points to TEE’s superiority in more accurately detecting and having a more profound impact on management strategies. At the same time, TTE has the benefits of being less invasive, more cost-effective, and widely available^[5]^. Still, a superiority trial discovered that TEE yielded more treatment-relevant findings than TTE^[6]^. TEE is gaining more ground among healthcare providers, given that it offers more treatment-altering findings, especially in stroke patients with unrecognized sources and more in younger groups^[7]^. Although TEE has led to increased detection of cardioembolic sources, it is essential to consider whether these findings translate to changes in the clinical management of patients. Several studies have yielded controversial results regarding TEE’s ability to guide secondary treatment approaches^[8-11]^. We aimed to collect data from patients who underwent a TEE study performed for the sake of a recent stroke diagnosis workup. We retrospectively analyzed the frequency of potential therapy-relevant findings on TEE in patients with acute ischemic stroke at the study institution and how often these findings led to a significant change in the treatment plan.

## Materials and methods

The study was conducted under the Strengthening the Reporting of Observational Studies in Epidemiology (STROBE) guidelines^[12]^. We adhered to the observational cohort guidelines, and our organization’s Institutional Review Board (IRB) reviewed the study protocol. Due to the study’s retrospective nature, it provided patient consent and waivers under the Health Insurance Portability and Accountability Act (HIPAA) (Study ID: Pro2022- 0633). The study was conducted in accordance with the Good Clinical Practice guidelines and the Declaration of Helsinki. It has been reported in line with the STROCSS criteria^[13]^.

Study design and setting: We conducted a retrospective, single-center cohort study between June 2021 and August 2022. The study location was a tertiary referral comprehensive stroke center in New Jersey State, USA, with a 691-bed capacity and a dedicated 13-bed neurointensive care unit.

### Participants

We obtained a list of all the patients who underwent TEE at our institution during the study period. We screened the indications for performing the study and included patients with acute ischemic stroke. All patients had an acute stroke confirmed by computed tomography (CT) or magnetic resonance (MRI) brain imaging. We included patients admitted to our hospital through the emergency department (ED) or transferred from a referral hospital. The transfer was due to a higher level of care, including neurovascular services in neurological intensive care units unavailable in nearby smaller hospitals. Researchers excluded the following: patients younger than 16 years, patients with no confirmed acute stroke, as seen on the CT or MRI scan, patients with a transient ischemic attack (TIA), and patients with other types of intracranial hemorrhage that is irrelevant to atherosclerosis or embolism, such as subdural hematoma or subarachnoid hemorrhage. We included all patients with missing patient characteristics or hospital-course information.

We included high-risk features based on guidelines for PFO and aortic atherosclerotic lesions. These features included a large PFO with a height of more than 2 mm, a long PFO (length of more than 10 mm), an atrial septal aneurysm, or a hypermobile atrial septum^[14]^.

### Outcomes

Our primary outcome was the detection of management changes based on TEE results. Secondary outcomes included positive TEE findings relevant to stroke, the performance of TEE as an inpatient versus outpatient procedure, the interval (in days) between the stroke diagnosis and TEE performance, and high-risk feature detection. We included patients who underwent transthoracic cardiac ultrasound before TEE and examined how this impacted the clinical course. Management changes were not considered or counted if they referred to a change in antiplatelet medications or other irrelevant medication classes, as these do not rely on cardiac imaging, which is the scope of the study. Furthermore, such changes depend solely on the provider’s decision for stroke prevention, regardless of cardiac structural findings on TEE.

### Variables and data sources

Clinical information was obtained from patients’ electronic medical records (EMRs) in the Epic system (Epic Systems Corporation, Verona, WI). Physicians collected and validated all data through manual chart review. The data collected included patient demographics, comorbidities, cardiovascular risk factors, physical examination findings, home medications, imaging findings, electrocardiograms (EKGs), and telemetry strips.

### Sample size calculation

We included all the patients admitted to our institution during the study period.

### Management of quantitative variables

We did not perform any binning of the quantitative variables. The interval between stroke and TEE indicated the number of days between the new diagnosis of acute/subacute stroke (by considering radiology report of CT or MR brain) and the day TEE was performed, regardless of whether it was done in inpatient or outpatient settings.

### Statistical analysis

Categorical variables were described as frequencies and percentages, while continuous variables were documented as mean values, standard deviations, and 95% confidence intervals (95% CIs). We used the chi-square test to compare categorical variables and the *t*-test with mean differences to compare continuous non-parametric variables. The odds ratio and the mean difference were used to determine the risk factors between the two groups. All calculations were performed using STATA 18.0 statistical software (StataCorp LLC, College Station, TX, USA), with an alpha value (p) 0.05 to determine statistical significance.

## Results

During the study period, 176 patients met the study inclusion criteria. We found that 66 patients (37.5% of the whole study population) had one or more abnormal findings that were considered to be related to an embolic stroke risk. Despite these findings, not all patients with positive TEE had a change in management based on the provider’s decision. For age and sex, there were no statistically significant differences between the positive and negative TEE groups. Notably, among stroke risk factors, hypertension (HTN) and smoking were the only factors that were statistically significantly higher in the negative TEE group. Fourteen patients in the negative TEE group and five patients in the positive TEE group were already on anticoagulation (AC) for a history of atrial fibrillation, deep venous thrombosis (DVT), recurrent stroke, or cardiac mural thrombus history. However, TEE led to a change in management, with three out of the five patients on existing AC being found to have PFO; subsequently, two underwent PFO closure as inpatients, and one planned for outpatient closure. The findings included 39 patients with PFO (22.2% of the whole study population), 11 patients with atherosclerotic plaque in the aorta (6.25% of the entire study population), six patients with valve, chamber, or device disease related to stroke (3.41% of the whole study population), and four patients with interatrial septal defect or aneurysm without PFO (2.27% of the entire study population). Additionally, there were six cases with multiple pathological findings (3.41% of the study population) (Table 1).

**Table 1:** Comparison of patients with TEE-positive findings relevant to stroke risk versus patients with no such findings.

Table1	Positive TEE	%	Negative TEE	%	*P*-value	test used	Test measure (chi2 has a chi-value, *t*-test has a *t*-value)
Number Total	66	37.5 % of total study population	110	62.5% of the total study population			
Age in years	65.3 SD 15.3 (95% CI: 61.5−69.05)		66.2 SD 15.7 (95% CI: 63.21–69.14)	Mean difference: 0.88 SD2.4(95% CI: − 5.67−3.9)	0.7153	Two-sample *t*-test with equal variances	0.3653
Female	31	47 %	50	45.5%	0.845	Chi2	0.0381
Race (patients)	66		108^&^		Percentages only for a total number of patients with specified race		
white 1	63	95.5%	75	69.4%	0.001 (for all races)	Chi2	17.2068
AA 2	2	3.0%	20	18.5%		Chi2	
Hispanic 3	0	0.0%	7	6.5%		Chi2	
Asian 4	1	1.5%	6	5.6%		Chi2	
Hypertension	39	59.1%	84	76.4%	0.016		5.8478
HLD	40	60.6%	64	58.2%	0.751		0.1003
DM	17	25.8%	39	35.5%	0.181		1.7879
(Number of patients with available data about LOS)	58^&^		100^&^				
LOS	9.9 SD 7.35 (95% CI: 7.98–11.85)		8.84 SD 6.78 (95% CI: 7.49–10.16)	Mean difference: 1.07 SD 1.15 (95% CI: − 1.21−3.35)	0.3536	Two-sample *t*-test with equal variances	−0.9304
Afib	5	7.6%	11	10.0%	0.588	Chi2	0.2933
CHF	6	9.1%	13	11.8%	0.572	Chi2	0.3186
CAD	13	19.7%	27	24.5%	0.457	Chi2	0.5522
History of previous stroke	14	21.2%	25	22.7%	0.815	Chi2	0.0549
Smoking	38	57.6%	37	33.6%	0.002	Chi2	9.667
Antiplat AD	27	40.9%	42	38.2%	0.72	Chi2	0.1287
Anticoag AD	5	7.6%	14	12.7%	0.286	Chi2	1.1368
Ischemic or hemorrhagic stroke	64^&^		105^&^		0.523	Chi2	1.2975
Ischemic	61	95.3%	99	94.3%			
Hemorrhagic	3	4.7%	2	1.9%			
Both	0	0.0%	4	3.8%			
TEE result with a finding as a potential embolic source*	66		110			Chi2	170
1	0	0.0%	110	100.0%			
2	39	59.1%	0	0.0%			
2,4	2	3.0%	0	0.0%			
2,5	1	1.5%	0	0.0%			
3	4	6.1%	0	0.0%			
4	11	16.7%	0	0.0%			
4,5	3	4.5%	0	0.0%			
5	6	9.1%	0	0.0%			

SD, standard deviation; AD, Afib, atrial fibrillation; Antiplatelet, antiplatelet use before admission; anticoagulant use before admission; LOS, length of stay; EF%, ejection fraction; CAD = coronary artery disease; CHF, congestive heart failure; DM = diabetes mellitus, HTN, hypertension; DVT, deep venous thrombosis; PE, pulmonary embolism; HLD, hyperlipidemia; LOS, length of stay; AA, African American

* (TEE findings: 1 = normal or no findings related to stroke occurrence; 2 = PFO; 3 = interatrial septal defects or aneurysm without PFO; 4 = aortic atherosclerotic disease excluding mild disease considered normal; 5 = valve, chamber, or device-related disease with embolic source).

& = missing patient information due to incomplete chart documentation.

### Impact of TEE findings on management

**Figure 1.**
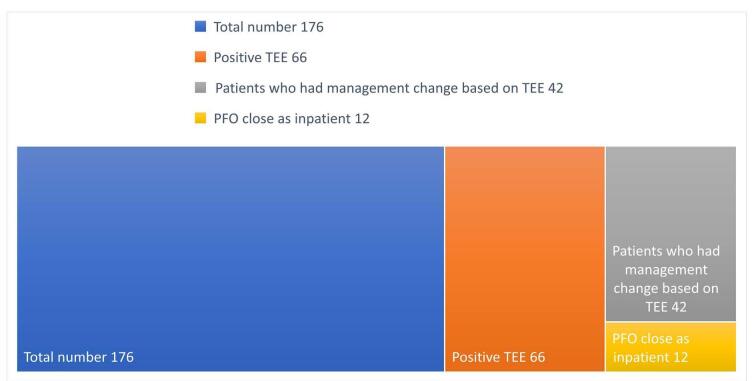
Diagram showing the proportion of patients in numbers with positive (abnormal) TEE findings and how much the change in management per study population; PFO close was the most common procedure performed as an inpatient.

The influence of TEE findings on management was as follows: Five (4.5%) patients with negative TEE results had a clinical management change based on the clinician’s discretion, with four of these patients either started on a direct oral anticoagulant (DOAC) or changed from one agent to another within the same DOAC category; in contrast, one patient was started on warfarin. In these cases, the provider decided to consider the patient’s risk factors for secondary prevention. On the other hand, positive or abnormal TEE results were found in 66 patients, with 42 patients (63.3%) experiencing a management change that included PFO closure during hospitalization in 12 patients (28.6%) (Fig. 1), a plan for PFO closure after discharge in 18 (42.9%), initiation or change of one DOAC agent to another in 15 (35.7%), and initiation of warfarin in one patient (2.38%). Additionally, one patient underwent pacemaker extraction with aortic valve replacement and ligation of the left atrial appendage, all in one patient. The total number of changes was 48, which is higher than the number of patients, 42, as some patients had more than one change; for example, some patients were planned for PFO closure and the start of DOAC simultaneously. The study population was divided into two groups based on age (less than 65 years versus more than 65 years) to study the effect of age in our population. However, there was no statistically significant change in management based on TEE findings according to age. Therefore, we used the mean difference measure of association between age and management change to find a correlation with the age factor, in contrast to a *t*-test with the odds ratio. We found a − 9.761905 mean age difference (in years) in the group with abnormal TEE findings compared to the other group (95% CI −17.26 to −2.26; *P*-value 0.0116). Age was a factor in TEE-based management change, with a higher chance of management change at a younger age.

Furthermore, female patients were more likely to get management change based on TEE findings. Additionally, patients with CAD (coronary artery disease) and CHF (congestive heart failure) history had a lower chance of changing management based on TEE findings, indicating less benefit from TEE (Table 2).

**Table 2:** Table 2: Impact of TEE findings on stroke patient management.

Table 2	OR	95% CI	*P*-value	
Age ≥ 65 years old	0.4985	0.1710 to 1.4526	0.202	Control* < 65
Sex (female)	3.2381	1.1090 to 9.4551	0.0316	
Race				
White	REF			
AA	0.5366	0.0320 to 9.0008	0.6652	
Hispanic	0.1807	0.0071 to 4.6216	0.3009	
Asian				
DM	0.7589	0.2449 to 2.3516	0.6326	
HTN	0.3333	0.1105 to 1.0057	0.0512	
HLD	0.8824	0.3147 to 2.4736	0.8119	
Afib	0.8462	0.1312 to 5.4572	0.8606	
CHF	0.0927	0.0101 to 0.8490	0.0353	
CAD	0.2703	0.0765 to 0.9546	0.0421	
History of previous stroke	0.3333	0.0993 to 1.1193	0.0755	
Smoking	0.4118	0.1415 to 1.1981	0.1034	
Antiplatelets	0.5556	0.2005 to 1.5393	0.2583	
Anticoagulants	7.1867	0.3801 to 135.8798	0.1885	
Atherosclerotic plaque with high-risk features any (sum) vs REF 1	0.8889	0.0613 to 12.8855	0.9312	
PFO risk features any (sum) vs 1	1	0.0419 to 23.8417	1	

Comparison of patients who underwent management changes based on TEE versus no management changes. OR = odds ratio. * = comparison between patient groups aged ≥ 65 years and those aged < 65 years.

Finally, six patients had more than one pathological finding. As we included all patients with PFO (including those with multiple findings), we identified 42 patients. All patients with aortic atherosclerotic disease (AortAth) were 16 (including those with numerous findings). Some PFO and AortAth patients had high-risk features (explained definitions in the methods), as follows: 1) PFO patients with moderate to large, long tunneled, atrial septal defect, and hypermobile septal aneurysm, 28 (66.6%) out of 42 patients with PFO; 2) patients with interatrial septal shunt or aneurysm without PFO who eventually did not undergo any change in management. Of the 16 patients with AortAth, 13 (81.3%) had high-risk features. Consequently, only three (23%) out of the 16 AortAth patients experienced a management change (in the form of DOAC initiation or change from one agent to another).

In conclusion, high-risk features of PFO or aortic atherosclerotic disease did not correlate significantly with management changes. Based on this, we can explain that providers did not rely solely on high-risk features in these categories; they likely considered the entire patient picture when deciding on changes. Additionally, providers implement no strict guidelines, leading to inconsistency in decisions regarding management changes, such as PFO closure as an inpatient or outpatient or a change in the anticoagulation management plan. Nevertheless, the PFO group had the highest number of patients in the positive TEE group and the highest number of patients with management changes compared to the other groups. Cardiologists sometimes proceeded with PFO repair, even for those without high-risk PFO features.

### Yield of TTE (transthoracic echocardiogram)

**Figure 2.**
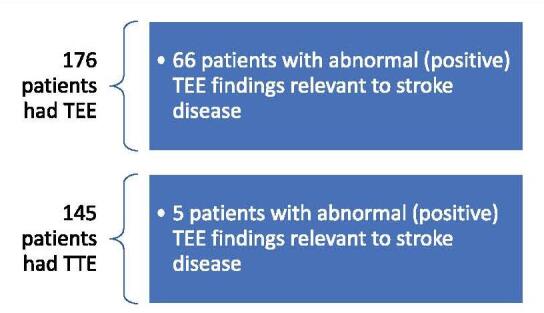
Yield of TTE (transthoracic echocardiogram) versus TEE (transesophageal echocardiogram).

Only five (3.4%) out of 145 patients in this study exhibited positive findings that might help stroke patients. The TTE yield was much lower than TEE’s (Table 3, Fig. 2). We identified only 145 out of 176 patients with available TTE records. The missing records were due to patients from other inaccessible facilities who had already carried out the TTE at their facility a few months before admission to our hospital. Hence, the provider did not want to repeat it. Another reason was that the provider decided to proceed directly to TEE because of a delay in TTE availability within the hospital. In some cases, the transthoracic module findings did not concur with those of the transesophageal counterpart, indicating that the TEE study was more accurate. All five patients with positive TTE findings had a management change due to the TEE results; there was no sole dependence on TTE in guiding management.

**Table 3:** TTE impact on management.

	Positive TTE with any abnormal findings (patient number)		Negative TTE with no abnormal findings (patient number)	
TTE if any findings relevant to stroke diagnosis patients	52^&^		93^&^	
1	47	90.4%	93	100.0%
2	0		0	
2 and 3	1	1.9%	0	0.0%
3 vegetations suspected	1	1.9%	0	0.0%
4 mural thrombus suspected	3	5.8%	0	0.0%

1 = no findings considered as stroke embolic source; 2 = interatrial aneurysm; 3 = interatrial defect; 4 = PFO

& = missing patient information due to incomplete chart documentation.

### TEE inpatient versus outpatient

Patients who had TEEs as OPs (outpatients) after hospital discharge were 38 (21.6%). Expectedly, their length of stay (LOS) was lower than those with IP (inpatient) TEE, with a mean difference of 5.7 days (*P* = 0.0001). We followed the patients via chart review, and none of them experienced another stroke after discharge until the TEE test day. The interval between stroke diagnosis and the TEE performance day was longer in outpatients, with a mean difference of 40 days (Table 4).

**Table 4:** Comparison of inpatient versus outpatient TEE.

	Inpatient TEE		Outpatient TEE		
Number	138	78.4%	38	21.6%	
LOS	10.2 SD 7.2 (95% CI: 8.93–11.4)		4.46 SD 1.9 (95% CI: 3.69–5.23)	Mean difference: 5.7 (95% CI: 2.88–8.5)	*P* = 0.0001
The interval between stroke diagnosis and TEE in days	3.97 SD 2.3 (95% CI: 3.58 –4.35)		44 SD 41.69 (95% CI: 29.22 –58.78)	Mean difference 40.03 (95% CI: − 47.0 to − 33.04)	*P* = 0

## Discussion

There is significant practical variability in the use of TEE to evaluate acute ischemic stroke, in part due to the need for established guidelines regarding appropriate patient selection. We found that one in three patients with acute ischemic stroke who underwent TEE had an abnormal stroke-relevant finding, with PFO being the most common pathology. Patient characteristics statistically significantly correlated with positive TEE results included race (across all races), hypertension, and smoking. Based on the TEE findings, the risk factors that led to a change in management were female patients with a history of coronary artery disease and a history of CHF (Table 1). Notably, patients with TEE abnormalities have lower rates of hypertension, possibly because uncontrolled blood pressure is a more potent risk factor for thrombotic (rather than embolic) stroke^[15,16]^. The use of TEE identified a possible cardiac source of embolism in 37.5% of acute ischemic stroke patients who underwent the procedure. This frequency is consistent with prior studies, which reported positive TEE findings in 11.1–49.0% of cases^[9,17]^. Similarly, our study substantiates that PFO was the most common finding, and it was detected in over 25% of ESUS and 4% of all stroke patients, according to a prior study^[18]^. While crucial evidence supports the ability of TEE to identify targets for secondary prevention, few studies to date have explored whether subsequent changes in therapeutic strategies occur due to these findings. Among patients with confirmed acute ischemic stroke who underwent TEE, 23.8% of them experienced a shift in medical management; 63.6% of the patients with new TEE findings experienced clinical management modification (Table 2). The productive yield of TEE in our study was slightly higher than that of other studies, which ranged from 7.1% of cases^[19]^ to 16.7%^[20]^. This variability may be explained by the small sample size of patients included and the potential institutional discrepancies in procedural volume rates of structural cardiac defects. Better published guidelines distributed among cardiologists might also explain why eligible patients were selected as physicians gained more experience. Our study showed that TTE could elaborate the source of cardiac emboli causing stroke, such as in cases of left ventricular thrombus, yet its yield is limited. It may be wise to skip TTE if the patient is already undergoing TEE. On a side note, there was a mismatch between TTE and TEE findings, with the latter showing a higher sensitivity and specificity (Table 3). Therefore, TEE is superior in detecting left atrial thrombus, spontaneous echo contrast, aortic valve atheroma, prosthetic valve abnormalities, atrial septal abnormalities, and cardiac tumors^[19]^.

In addition to assessing which patients are appropriate candidates for screening, it is essential to consider the ideal clinical setting for TEE. Despite patients waiting 34.5 days longer on average for TEE in the outpatient setting, no patient experienced another stroke event before imaging. This could counter the argument for hospital policies that mandate performing TEE before stroke patients get released from the hospital. Stroke recurrence is a significant concern for responsible physicians. According to Eriksson *et al*. (2020), stroke recurrence was 3.7% after 1 year, 7.0% after 3 years, and 9.1% after 5 years^[21]^. For patients who received inpatient TEE, there was an increased length of stay by approximately six additional days (mean difference of 5.7 days) (Table 4). Patients were required to complete their diagnostic workup before being cleared for discharge, including an echocardiogram (either transthoracic or transesophageal). For example, if scheduled during the weekend, the patient must wait until Monday. Using a conservative estimate of $500 (U.S. dollars) per hospital day^[22]^, there was a potential saving of around $280,000 during the study period if all 140 inpatient TEEs were deferred to outpatient status. Since there were no reported stroke recurrences during the intervals in our cohort from discharge to outpatient TEE, patients returning to outpatient TEE underwent a thorough examination before the procedure, with findings documented during the visit. This approach suggests that it is both safe and cost-effective without the risk of developing a new stroke. Regarding long-term patient outcomes, our study did not extend beyond the index admission for stroke. We followed up on 9 of 11 cases to check the TEE patient’s subsequent visit and found no event between admission and outpatient procedures. We confirmed any new admissions through hospital chart reviews and by reviewing the cardiologist’s brief history description in the TEE visit report.

## Limitations

Our study has some limitations. These include the single-institution study design, which contributed to the small sample size of patients. Additionally, relying on data from a single center may impose limitations when generalizing these findings due to institutional and geographic differences. Furthermore, the retrospective nature of this study could be associated with the inability to deduce causality. The retrospective approach of this study introduces selection bias. At that time, the cardiologist and neurologist decided to perform TEE. However, the patients included in our study had a sole indication for undergoing TEE, which was to try to determine the potential cardioembolic source of the stroke.

## Conclusions

Transesophageal echocardiography (TEE) identifies potential cardioembolic sources in patients with acute ischemic stroke, with patent foramen ovale (PFO) representing the most frequent finding. Most TEE abnormalities were treatment-relevant, leading to a significant change in clinical management in approximately two-thirds of patients with positive TEE findings and nearly a quarter of our study population. Our study proves that TEE is more accurate than TTE and confirms that TEE is highly effective in diagnosing potential cardioembolic sources, which can favorably affect secondary stroke prevention therapies. More studies are needed to clearly define how patients would benefit from management changes based on TEE findings. Furthermore, we found that TEE may be safely deferred in the short term to the outpatient setting without compromising patient safety or diagnostic yield while saving precious healthcare resources and reducing costs.

## Data Availability

Data are available to our hospital provider upon request only.

## Research registration unique identifying number (UIN)

Researchregistry.com, https://www.researchregistry.com/browse-registry#home/registrationdetails/66ab19444f9dc400277bb2cc/. The unique identifying number is researchregistry10551.
